# The related SNPs and genes to body size using GWAS- latent variable modeling in dromedaries

**DOI:** 10.1186/s12864-025-11766-9

**Published:** 2025-07-08

**Authors:** Morteza Bitaraf Sani, Morteza Mokhtari, Zahra Roudbari, Omid Karimi, Nader Asadzadeh, Faisal Almathen, Mohammad Hossein Banabazi

**Affiliations:** 1 Animal Science Research Department, Yazd Agricultural and Natural Resources Research and Education Center, Agricultural Research, Education & Extension Organization (AREEO), Yazd, Iran; 2 Department of Animal Science, Faculty of Agriculture, University of Jiroft, Jiroft, Iran; 3 Department of Animal Viral Diseases Research, Agricultural Research, Education and Extension Organization, Razi Vaccine and Serum Research Institute, Karaj, 3146618361 Iran; 4 Agricultural Research, Education and Extension Organization (AREEO), Animal Science Research Institute of Iran, Karaj, 3146618361 Iran; 5 Department of Veterinary Public Health and Animal Husbandry, College of Veterinary Medicine and Animal Resources, King Faisal University, 400 Al-Hasa, Hofuf, Saudi Arabia; 6 Department of Animal Breeding and Genetics (HGEN), Centre for Veterinary Medicine and Animal Science (VHC), Swedish University of Agricultural Sciences (SLU), Uppsala, 75007 Sweden

**Keywords:** Body measurements, Candidate genes, Factor analysis, Structural equation model

## Abstract

Camels are increasingly recognized for their potential to meet future nutritional and medical needs due to their unique qualities. This study aims to advance our understanding of the genetic basis of body size in dromedaries by employing confirmatory factor analysis (CFA) and genome-wide association studies (GWAS). We used phenotypic data from 9 body measurements of 96 Iranian male camels to develop a latent variable model for body size. The CFA model demonstrated excellent fit (CFI = 0.99, TLI = 0.99, RMSEA = 0.05, SRMR = 0.02), confirming that the selected biometric traits effectively capture the body size latent variable. Subsequent GWAS, utilizing 14,522 SNPs, identified 13 significant SNPs associated with body size across several chromosomes. The candidate genes linked to these SNPs, including *UBE3D*,* REPS1*,* SLC4A1AP*,* EFR3B*,* PRR11*, and *VMP1*, were further examined through Gene Ontology (GO) enrichment analysis, revealing their involvement in crucial biological processes such as catabolic and metabolic activities, developmental processes, and protein and lipid transport. These findings provide valuable insights into the genetic mechanisms underpinning body size in dromedaries, offering a foundation for future research and potential applications in breeding and genetic improvement strategies.

## Introduction

Camels hold significant promise for meeting future human food and medical needs due to their distinctive qualities. Their meat, milk, and other products are known to offer a range of health benefits [11]. Therefore, it is essential to explore their productive capabilities within their natural environments, as camels have a remarkable genetic potential compared to other ruminants [2]. Camel breeders primarily aim to enhance genetics to produce dual-purpose animals that are efficient in both meat and milk production [10].

The long generation interval of camels makes genetic improvement through traditional breeding methods a slow process, rendering significant advancements difficult to achieve using conventional techniques. Moreover, the costs associated with breeding through progeny testing are substantially higher in the traditional methods compared to advanced genomic-based approaches. As a result, considering challenges in recording and phenotyping camels, marker-assisted selection (MAS) offers a promising alternative for evaluating dromedaries in extensive production systems [3].

In genetic evaluation programs of livestock species, the more accurate evaluation of selection candidates is largely based on several traits [7, 21]. A computationally important feature of multivariate mixed models is that the number of parameters increases as more traits are included in the analysis, which can complicate the feasibility of genetic studies [26]. Structural equation modeling (SEM) is a powerful statistical technique, originally introduced by Wright, that enables the exploration of causal relationships among traits [30]. SEM applies various models to explain the relationships between observed variables and provides a framework for testing theoretical models representing these relationships [24]. This approach allows researchers to evaluate functional causal relationships between traits, making it possible to infer these relationships and also quantifying the strength of their associations [18].

Latent variable modeling (LVM) is a dimension reduction technique and a specific application of structural equation modeling (SEM) [24]. It can be used on phenotypic data to decrease data dimensionality and reduce computational complexity. According to Silva et al. [26], a small number of latent variables can effectively reduce data dimensionality, thus addressing the complexity caused by model over-parameterization. Although latent variables cannot be measured directly, they can be described by several observable variables [24]. LVM enables the investigation of complex phenomena by condensing multiple measurable traits into a few underlying latent variables [12]. Confirmatory factor analysis (CFA) is one statistical method that can be used to construct latent variables [31]. CFA is typically used to assess the hypothesis that a group of measurable variables corresponds to a smaller number of latent variables [23].

In a previous study, Bitaraf Sani et al. identified SNPs and genes associated with 12 morphometric traits in dromedaries using genotype-by-sequencing (GBS), a linear mixed model incorporating principal component analysis (PCA), and a kinship matrix [2]. The LVM can be applied as a dimension-reduction technique to the phenotypic data, for reducing the dimensionality of data and minimizing computational complexity. By leveraging the correlation between morphometric traits and animal performance, the CFA can be used for constructing latent variables based solely on morphometric measurements. Using latent variables allows researchers to understand the complexity of phenotypic traits, discover underlying structures among variables, model polygenic effects, and reduce the dimensionality of genomic data [12]. This study aimed to model and identify the latent trait of body size (BS) using 9 measurable body dimensions, to perform a genome-wide association study (GWAS) for BS, and to identify candidate genes in dromedaries in the central desert of Iran.

## Materials and methods

### Ethics and consent to participate

We obtained informed consent from the owner(s) to use the animals. Blood samples were collected during qualified veterinary treatment within the framework of governmental programs aimed at the animal identification, monitoring of health, and parentage confirmation of the dromedary populations included in our study. No other kind of tissue was used in this study. No anesthesia and euthanasia was done in the study.

### Investigated biometric traits

In this study, phenotypic records were used for nine body measurements of 96 Iranian male camels, as shown in Table 1. Among the 96 recorded camels, 18 camels belonged to the National Research and Development Station on Dromedary Camel (Bafgh), and the rest belonged to local herds in Ardakan, Bahabad, Mehriz, Khatam, and Bafagh cities. It provides a clear overview of the mean values and standard errors for each of the body measurements recorded. For further details about the animals and phenotypes, please refer to the study by Bitaraf Sani et al. [2].

**Table 1:** Summarizing the phenotypic records of the 9 body measurements for 96 Iranian male camels

Body Measurement	Mean (cm) ± SE
Head Length (HL)	33.27 ± 0.32
Muzzle Circumference (MC)	30.73 ± 0.29
Neck Length (NeL)	60.10 ± 0.93
Chest Circumference (ChC)	113.73 ± 1.77
Hump Height (HH)	134.28 ± 1.30
Body Length (BL)	84.75 ± 1.39
Tail Length (TL)	37.62 ± 0.40
Pin Width (PW)	26.48 ± 0.41
Abdominal Width (AW)	31.80 ± 0.36

### Latent variable modeling

**Fig. 1:**
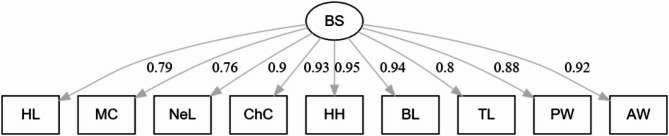
Representation of latent variable of BS and the respective relationships with the considered body measurement traits including Head Length (HL), Muzzle Circumference (MC), Neck Length (NeL), Chest Circumference (ChC), Hump Height (HH), Body Length (BL), Tail Length (TL), Pin Width (PW), and Abdominal Width (AW)

Figure 1 depicts the theoretical model considered to construct the body size (BS) latent variable from nine biometric traits. The CFA is applied to identify the latent factors that account for variations in BS. Conducting CFA requires establishing a measurement model that defines the relationships between observable variables and the latent factors. The CFA model used in this study is as follows:

In this model, ƺ denotes the vector of HL, MC, NeL, ChC, HH, BL, TL, PW, and AW measured variables. ξ represents the vector of latent factors. The matrix **Λ** contains the factor loadings that relate these latent factors to the measurable variables, while δ signifies the vector of residuals. The CFA model was fitted using the lavaan package [10] in the R environment [20]. The model’s overall fit was evaluated with four goodness-of-fit indices including the standardized root mean square residual (SRMR) [1], root mean square error of approximation (RMSEA) [27], TLI and CFI [1]. Additionally, the model was tested for bias through permutation using the bootstrap function from the lavaan package [22] with 5,000 bootstrap samples. The values of a latent variable are not directly measurable but are inferred from other measured variables by using statistical models [24].

### SNP data, GWAS analysis, identification of candidate genes, and gene ontology (GO) enrichment analysis

The study utilized SNP data from 96 Iranian male camels. More information on SNP data and genomic information is provided by Bitaraf Sani et al. [2]. Genotyping was conducted using genotype-by-sequencing (GBS) on the Illumina HiSeq 2000 platform, with the process performed by Bayan Gene Pars Company. This involved fragmenting DNA with the restriction enzymes *EcoR1* and *HinF1*, attaching adapters to the fragments, and amplifying them with DNA polymerase [2]. The sequence reads were mapped to the dromedary reference genome assembly on chromosome level (GCA_000803125.3 [1]; ) by using the BWA-MEM algorithm of Burrows–Wheeler Aligner (BWA) [15]. The GWAS analysis was carried out using TASSEL software version 5.2.48 [6]. After filtering for a minor allele frequency (MAF < 0.01), 14,522 SNPs were analyzed. The association between SNPs and body size (BS) was evaluated using the MLM_PCA + K statistical model, which included region as a fixed effect and camel age as a covariate. Data were collected during birth to 6 age-months old (every three months) from five regions of central desert of Iran in 2018. Suggestive significance thresholds for Bonferroni p-values were established at (− log p-value > 4) using the GEC software tool [14].

The Ensembl Variant Effect Predictor (VEP) web interface (http://www.ensembl.org/vep) was utilized for the annotation and prioritization of genomic variants located in both coding and non-coding regions [17]. It assesses the molecular consequences of variants by utilizing the Ensembl/GENCODE or RefSeq gene sets. Additionally, Ensembl VEP offers filtering options to customize variant prioritization. The tool is well-supported and is updated approximately every quarter to incorporate the most recent gene, variant, and phenotype association data. Gene Ontology (GO) provides a consistent framework for globally categorizing gene functions. In this study, we utilized GO to clarify the roles and functions of the identified genes by assigning them to three distinct ontologies: Molecular Function (MF), Cellular Component (CC), and Biological Process (BP). The analysis was conducted using the DAVID database [8, 25] and the web-based Gene Ontology Resources interface (http://geneontology.org/).

## Results

The findings from this study offer valuable insights into the body size (BS) latent variable in dromedaries. Through a combination of confirmatory factor analysis (CFA) and genome-wide association studies (GWAS), we have been able to investigate and characterize the genetic factors affecting body size in these animals. The CFA results confirmed that our measurement model accurately represents body size, with biometric traits effectively capturing this latent variable. Additionally, the GWAS pinpointed several significant SNPs linked to body size, demonstrating the role of various genetic factors across the genome in influencing this trait. This combined approach not only validates our measurement model but also reveals the intricate genetic mechanisms behind body size in dromedaries, providing a solid foundation for future research and practical applications in breeding and genetic studies.

### Extracting BS latent variable

Figure 1 illustrates the schematic diagram of the measurement model proposed for modeling the BS latent variable. The goodness-of-fit indices for the model were as follows: CFI = 0.99, TLI = 0.99, RMSEA = 0.05, and SRMR = 0.02. Table 2 presents the standardized factor loadings for the measurable variables including HL, MC, NeL, ChC, HH, BL, TL, PW, and AW, which were 0.79, 0.76, 0.90, 0.93, 0.95, 0.94, 0.80, 0.88, and 0.92, respectively. All factor loadings were statistically significant (*p* < 0.01). Positive factor loadings indicate that an increase in any of the measurable body dimension variables is associated with an increase in the BS latent variable. The goodness of fit for the measurement model was assessed using statistical indices, all of which demonstrated that the model fit the data exceptionally well. Furthermore, all factor loadings were positive and significantly different from zero (*p* < 0.01), confirming that the considered measurable variables of body dimensions appropriately represented the latent variable of BS in Iranian male dromedaries.

**Table 2:** Standardized factor loadings of the measurable body dimension traits used for describing BS latent variable in dromedaries

Trait	Factor loading	Standard error	Z-value
HL	0.79	0.02	35.02
MC	0.76	0.04	21.17
NeL	0.90	0.02	38.93
ChC	0.93	0.02	48.63
HH	0.95	0.01	111.60
BL	0.94	0.03	34.92
TL	0.80	0.03	29.45
PW	0.88	0.02	36.86
AW	0.92	0.01	64.75

### GWAS and significant SNPs

**Fig. 2:**
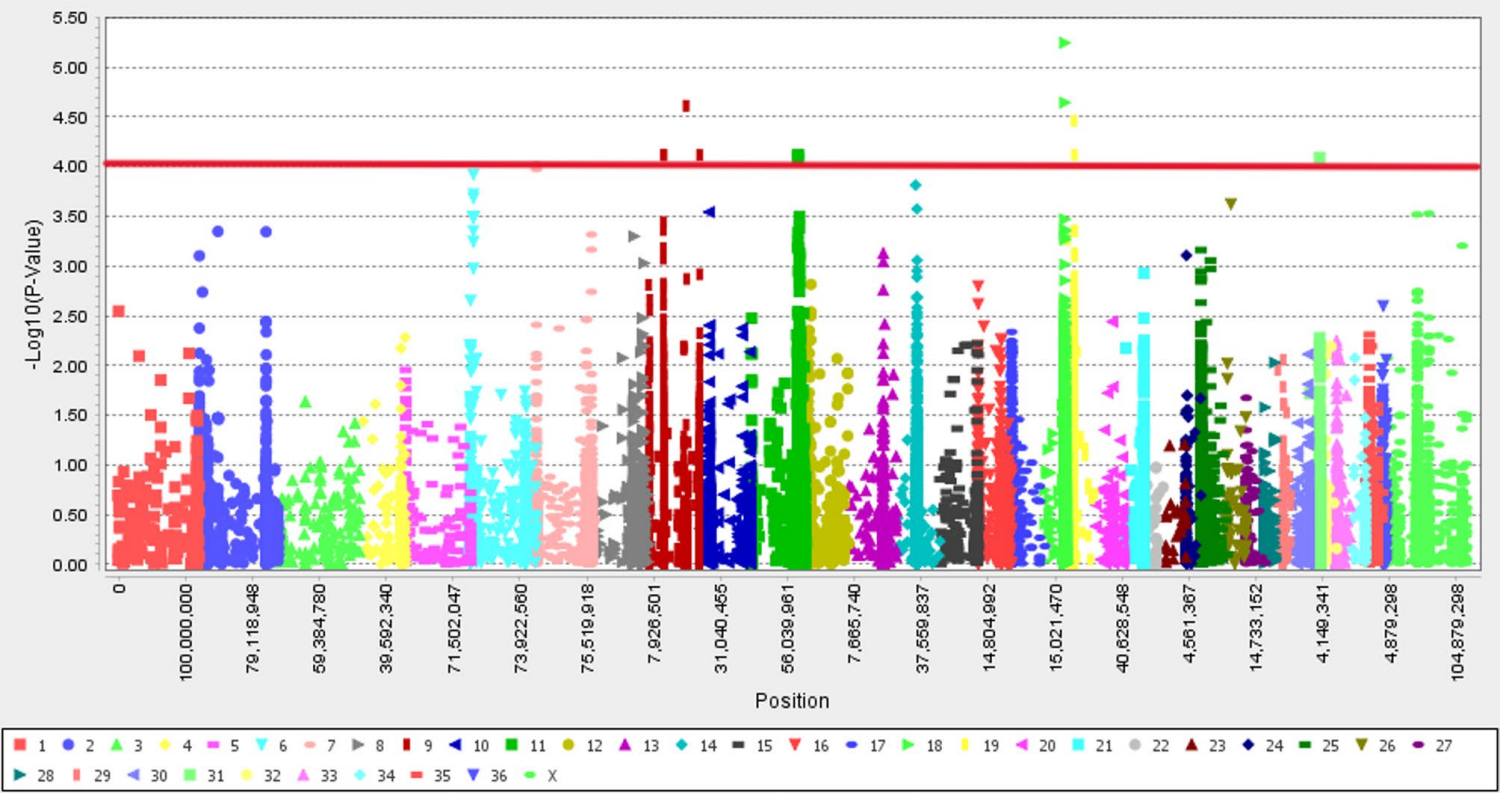
Manhattan plot representing chromosome-wide association with the body size latent trait in dromedaries using MLM_PCA + K GWAS. The red horizontal line represents the set significant threshold (–log10 *p* value = 4)

**Fig. 3:**
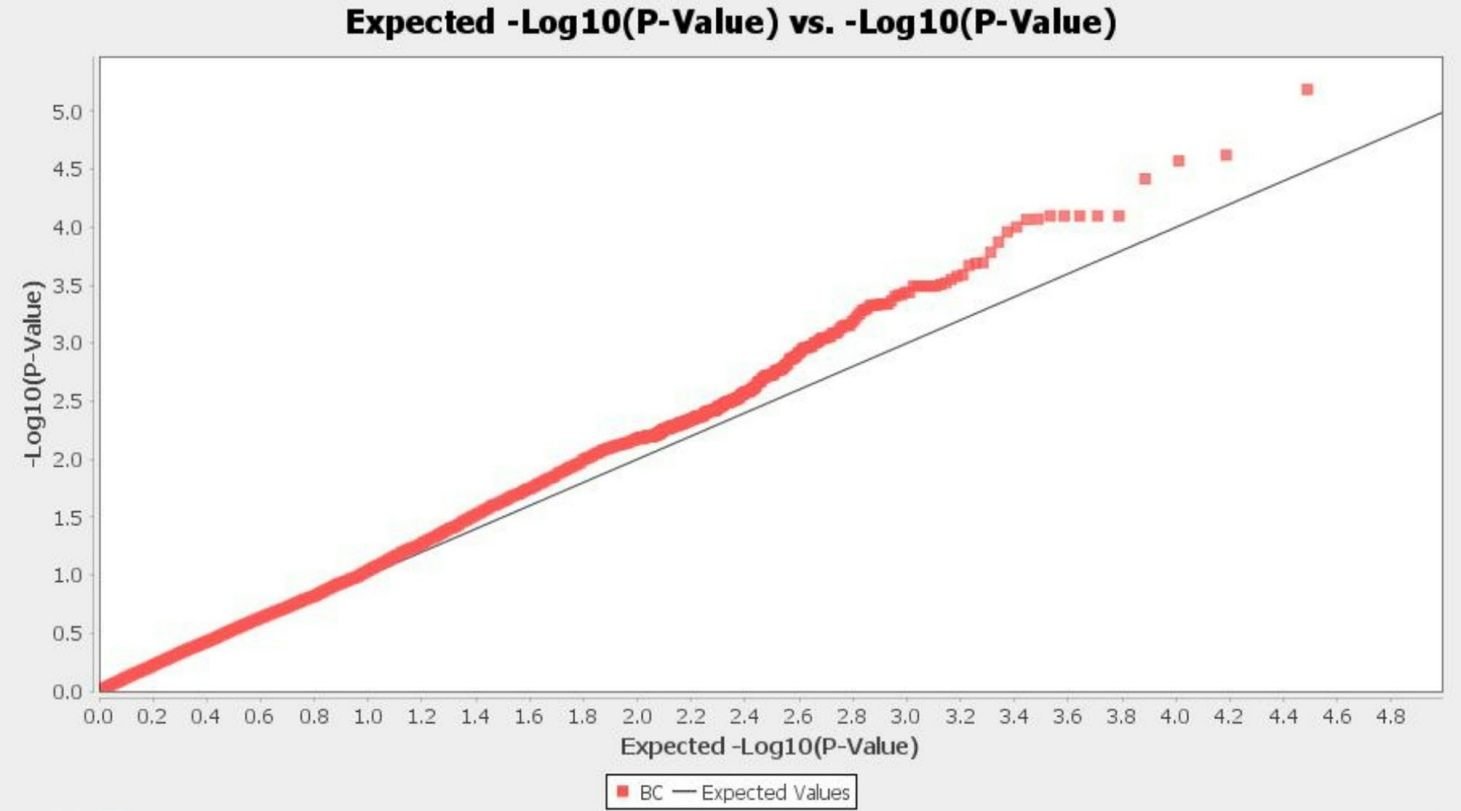
Q-Q plot displays GWAS results from TASSEL for the body size latent trait in dromedaries

In this study, a genome-wide association study (GWAS) was performed with 14,522 SNPs, revealing 13 SNPs linked to the body size latent variable on chromosomes 7, 9, 11, 14, 18, 19, and 31 (refer to Figs. 2 and 3, and Table 3). SNPs influencing body size are dispersed across the genome [16], with numerous genes potentially involved in regulating this trait [5]. By contrast, Banestani et al. [23] identified 53 significant SNPs associated with body size in pigs using GBLUP-GWAS. The six genes including *UBE3D*,* REPS1*,* SLC4A1AP*,* EFR3B*,* PRR11*, and *VMP1* in the flanking regions of top SNPs (see Table 3) were proposed as candidate genes related to the BS latent variable in dromedaries.

**Table 3:** Associated SNPs and genes for body size latent trait in dromedary

SNP	Chromosome	Position	-Log ( *P*-Value)	Candidate Gene
Chr7_208342	7	208,342	4.00	*-*
Chr9_56195360	9	56,195,360	5.20	*-*
Chr9_22874300	9	22,874,300	4.10	*UBE3D*
Chr9_76680599	9	76,680,599	4.10	*REPS1*
Chr11_71991797	11	71,991,797	4.10	*SLC4A1AP*
Chr11_71991805	11	71,991,805	4.10	*SLC4A1AP*
Chr11_74157812	11	74,157,812	4.09	*EFR3B*
Chr11_71991811	11	71,991,811	4.07	*SLC4A1AP*
Chr14_29144338	14	29,144,338	4.47	*-*
Chr18_29891428	18	29,891,428	4.27	*-*
Chr19_10153908	19	10,153,908	4.42	*PRR11*
Chr19_10684174	19	10,684,174	4.10	*VMP1*
Chr31_16992831	31	16,992,831	4.07	*-*

### Enrichment analysis for candidate genes

In our investigation, we conducted a Gene Ontology (GO) enrichment analysis on six candidate genes linked to body size. This analysis provided valuable insights into the roles of these genes across various biological dimensions (Table 4). Notably, five of these genes were found to be significantly enriched in diverse biological processes. These include catabolic and metabolic activities, which are fundamental for the breakdown and utilization of substances within the organism. Additionally, these genes play critical roles in biological and developmental processes, which are essential for growth and overall development. The enrichment in anatomical structure development and multicellular organism development further underscores their involvement in shaping and maintaining complex body structures.

Furthermore, the analysis highlighted four of these genes as being enriched in molecular functions. These functions encompass vital roles such as protein and lipid transport and lipid translocation, which are crucial for maintaining cellular and systemic balance. The ability of these genes to facilitate these processes suggests their importance in regulating and supporting bodily functions.

Additionally, all six candidate genes were significantly enriched in various cellular components. This enrichment indicates that these genes are integral to essential cellular structures and processes, reinforcing their importance in maintaining cellular integrity and function. Table 4 provides a comprehensive summary of these findings, demonstrating the intricate roles these genes play in the regulation of body size. Overall, this GO enrichment analysis underscores the complex interplay of genetic factors involved in body size regulation, offering a deeper understanding of the genetic mechanisms underpinning growth and development in dromedaries.

**Table 4:** Candidate genes significantly enriched to the body size latent trait-related GO terms in dromedaries

Enrich type	Genes	Term
BP	EFR3B	GO:0046854 ~ phosphatidylinositol phosphate biosynthetic process, GO:0072659 ~ protein localization to the plasma membrane
	REPS1	GO:0006897 ~ endocytosis, GO:0016197 ~ endosomal transport, GO:0150007 ~ clathrin-dependent synaptic vesicle endocytosis
	SLC4A1AP	GO:0000398 ~ mRNA splicing, via spliceosome, GO:0035196 ~ miRNA processing, GO:0035308 ~ negative regulation of protein dephosphorylation, GO:0051237 ~ maintenance of RNA location,
	UBE3D	GO:0009056 ~ catabolic process, GO:0008152 ~ metabolic process, GO:0008150 ~ biological process, GO:0000209 ~ protein polyubiquitination, GO:0006513 ~ protein monoubiquitination, GO:0019538 ~ protein metabolic process, GO:0030163 ~ protein catabolic process, GO:0036211 ~ protein modification process
	VMP1	GO:0009056 ~ catabolic process, GO:0008152 ~ metabolic process, GO:0008150 ~ biological process, GO:0034329 ~ cell junction assembly, GO:0042953 ~ lipoprotein transport, GO:0098609 ~ cell-cell adhesion, GO:0140056 ~ organelle localization by membrane tethering, GO:1,901,896 ~ positive regulation of ATPase-coupled calcium transmembrane transporter activity, GO:1,990,456 ~ mitochondrion-endoplasmic reticulum membrane tethering, GO:0032502 ~ developmental process, GO:0048856 ~ anatomical structure development, GO:0007275 ~ multicellular organism development, GO:0015031 ~ protein transport, GO:0006869 ~ lipid transport, GO:0034204 ~ lipid translocation
MF	REPS1	GO:0005509 ~ calcium ion binding, GO:0005515 ~ protein binding, GO:0017124 ~ SH3 domain binding, GO:0060090 ~ molecular adaptor activity
	SLC4A1AP	GO:0003729 ~ mRNA binding, GO:0004865 ~ protein serine/threonine phosphatase inhibitor activity, GO:0005515 ~ protein binding
	UBE3D	GO:0030332 ~ cyclin binding, GO:0031624 ~ ubiquitin conjugating enzyme binding, GO:0061630 ~ ubiquitin protein ligase activity
	VMP1	GO:0017128 ~ phospholipid scramblase activity
CC	EFR3B	GO:0005829 ~ cytosol, GO:0005886 ~ plasma membrane, GO:0015629 ~ actin cytoskeleton
	REPS1	GO:0005654 ~ nucleoplasm, GO:0005737 ~ cytoplasm, GO:0005829 ~ cytosol, GO:0005886 ~ plasma membrane, GO:0005905 ~ clathrin-coated pit, GO:0042734 ~ presynaptic membrane, GO:0097708 ~ intracellular vesicle
	PRR11	GO:0005634 ~ nucleus, GO:0005737 ~ cytoplasm
	SLC4A1AP	GO:0005886 ~ plasma membrane, GO:0016607 ~ nuclear speck
	UBE3D	GO:0000151 ~ ubiquitin ligase complex, GO:0005634 ~ nucleus, GO:0005829 ~ cytosol
	VMP1	GO:0000421 ~ autophagosome membrane, GO:0005783 ~ endoplasmic reticulum, GO:0005789 ~ endoplasmic reticulum membrane, GO:0005886 ~ plasma membrane, GO:0012505 ~ endomembrane system, GO:0016020 ~ membrane, GO:0033116 ~ endoplasmic reticulum-Golgi intermediate compartment membrane

## Discussion

### BS latent variable

In this study, the goodness-of-fit measures showed that the proposed measurement model for the BS latent variable is well-suited. Similar approaches have been used in previous research involving latent variables in livestock species. For example, Penagaricano et al. [18] analyzed 19 phenotypic traits in pigs and identified five latent variables. Leal-Gutierrez et al. [12] defined a latent variable for carcass quality in beef cattle using variables such as quality grade, fat over ribeye, and marbling. Silva et al. [26] used 14 traits in broilers to develop four latent variables through Bayesian confirmatory factor analysis (CFA). Sanjari Banestani et al. [23] established a latent variable for body size in Yorkshire pigs using five observed body measurements including body length, body height, chest width, chest girth, and tube girth.

### Candidate genes

*UBE3D* and *REPS1* play crucial roles in growth and cellular signaling pathways. *UBE3D*, involved in mRNA 3’-end processing, regulates gene expression essential for cellular differentiation and adipogenesis. Its activity ensures proper mRNA processing, maintaining the adipocyte-committed state, and supporting growth through differentiation [9]. REPS1, a Rab5 effector, is integral to endocytosis and recycling processes, including the transferrin receptor pathway. It facilitates nutrient uptake and receptor recycling, vital for cell growth and signaling. Both genes contribute to growth by regulating critical cellular processes and signaling pathways, ensuring proper cellular function and development [13]. *SLC4A1AP* and *EFR3B* are essential for cellular function and development through their roles in ion transport and membrane stability. *SLC4A1AP* is involved in maintaining optimal pH and ionic balance, which is crucial for metabolic processes. It works alongside AE1 (anion exchanger 1) to facilitate efficient ion transport, ensuring that cells can regulate their internal environment effectively. Disruptions in this transport can lead to metabolic dysfunction and developmental abnormalities [19]. EFR3B contributes to membrane stability and lipid synthesis, supporting the structural integrity of cells. It is crucial for the recruitment of the phosphatidylinositol 4-kinase (*PI4KIIIα*) complex to the plasma membrane, which is vital for lipid composition and signaling pathways [28]. *EFR3B’s* role in modulating G-protein-coupled receptor responsiveness further emphasizes its importance in cellular signaling [4]. *PRR11* and *VMP1* play significant roles in tissue growth and maintenance by regulating critical cellular processes. *PRR11* is implicated in cell cycle progression, particularly in the transition from late S phase to G2/M phase. It has been shown to induce premature chromatin condensation, which is essential for accurate cell division. This regulation of the cell cycle is crucial for tissue growth and development, as it ensures proper cellular proliferation. *VMP1* is a transmembrane protein that triggers autophagy, a process vital for cellular homeostasis and the degradation of damaged organelles. VMP1 also plays a role in regulating calcium homeostasis within the endoplasmic reticulum, which is important for various cellular functions, including signaling pathways [29].

## Conclusion

This research provides important information on the genetic basis of BS latent variable in Iranian male dromedaries by integrating confirmatory factor analysis (CFA) with genome-wide association studies (GWAS). By applying a latent variable model, we accurately described BS using nine measurable body dimension variables from 96 male Iranian camels. The CFA confirmed the reliability of our model, showing that the considered nine measurable body dimension variables effectively represent the BS latent variable. The GWAS revealed 13 significant SNPs linked to BS across several chromosomes. Key candidate genes such as *UBE3D*,* REPS1*,* SLC4A1AP*,* EFR3B*,* PRR11*, and *VMP1* were associated with these SNPs and were involved in crucial biological processes, including catabolic and metabolic functions, development, anatomical structure formation, and cellular transport. Gene Ontology (GO) enrichment analysis highlighted the significant roles of these genes in genetic regulation of BS through various biological functions. This study promotes our understanding of the genetic framework influencing BS in dromedaries and paves the way for future genetic improvement and marker-assisted selection in camel breeding. The combination of CFA and GWAS methodologies provides a thorough approach to deciphering the complex genetic determinants of body size, offering valuable insights into animal genetics and breeding.

## Data Availability

The variant data for this study have been deposited in the European Variation Archive (EVA) at EMBL-EBI under accession number PRJEB82242 (https://www.ebi.ac.uk/eva/?eva-study=PRJEB82242); public on 2024-11-09.
